# Molecular Detection of *Streptococcus downii* sp. nov. from Dental Plaque Samples from Patients with Down Syndrome and Non-Syndromic Individuals

**DOI:** 10.3390/microorganisms10061098

**Published:** 2022-05-26

**Authors:** Eliane García-Mato, Lucía Martínez-Lamas, Maximiliano Álvarez-Fernández, Iván Varela-Aneiros, Marcio Diniz-Freitas, Jacobo Limeres-Posse, Pedro Diz-Dios

**Affiliations:** 1 Medical-Surgical Dentistry Research Group (OMEQUI), Health Research Institute of Santiago de Compostela (IDIS), University of Santiago de Compostela (USC), 15705 Santiago de Compostela, Spain; eliane.garma@gmail.com (E.G.-M.); ivan.varela.aneiros@gmail.com (I.V.-A.); jacobo.limeres@usc.es (J.L.-P.); 2 Clinical Microbiology, Microbiology and Infectiology Group, Galicia Sur Health Research Institute, Hospital Álvaro Cunqueiro, Complejo Hospitalario Universitario de Vigo, 36312 Vigo, Spain; lucia.martinez.lamas@sergas.es (L.M.-L.); maximiliano.alvarez.fernandez@sergas.es (M.Á.-F.)

**Keywords:** *Streptococcus downii*, Down syndrome, *Streptococcus mutans*, caries, periodontal disease, oral health

## Abstract

A new bacterial species has recently been identified in the dental plaque of an adolescent with Down syndrome. The species is known as *Streptococcus downii* sp. nov. (abbreviated to *S. downii*), and it inhibits the growth of *S. mutans* and certain periodontal pathogens. The aim of this study was to determine the distribution of *S. downii* in the oral cavity of individuals with Down syndrome. **Methods**: A specific polymerase chain reaction for the operon of bacteriocin (class IIb lactobin A/cerein 7B family) was designed to detect *S. downii* in individuals with Down syndrome (*n* = 200) and in the general population (*n* = 100). We also compared the whole genome of *S. downii* and the regions related to its bacteriocins against 127 metagenomes of supragingival plaque of the “Human Microbiome Project”. **Results**: We detected the specific gene of the *S. downii* bacteriocin in an individual with Down syndrome (Cq, 34.52; GE/μL, 13.0) and in an individual of the non-syndromic control group (Cq, 34.78 Cq; GE/μL, 4.93). The prevalence of *S. downii* was ≤1% both in Down syndrome and in the general population, which did not allow for clinical-microbiological correlations to be established. This result was confirmed by detecting only one metagenome with an ANIm with approximately 95% homology and with 100% homology with ORFs that code class IIb lactobiocin A/cerein 7B bacteriocins among the 127 metagenomes of the “Human Microbiome Project” tested. **Conclusions**: The detection rate of *S. downii* in the supragingival dental plaque was very low, both in the Down syndrome individuals and in the non-syndromic controls. A clinical-microbiological correlation could therefore not be established.

## 1. Introduction

Dental caries is the most prevalent non-transmittable disease globally, given the estimated 2.3 billion individuals with caries in their permanent teeth and more than 530 million children with caries in their temporary teeth [1]. Although genetic, microbial, immunological, behavioral and environmental factors are involved in the development of caries, massive sequencing techniques have helped establish that caries is an expression of dysbiosis of the oral microbiome [2]. However, it has classically been considered that the fundamental etiological agent is a complex polymicrobial community in which *Streptococcus mutans* has an especially relevant role [3]. Paradoxically, a number of bacterial species have been identified that inhibit the growth of *S. mutans*, and participate in the homeostasis of the oral biofilm and consequently exhibit anticaries potential. These species include certain hydrogen peroxide-producing streptococci such as *S. oligofermentans* [4] and *S. A12* [5]; arginolytic streptococci such as *S. gordonii*, *S. sanguinis*, *S. parasanguinis*, *S. australis* and *S. cristatus* [6]; bacteriocin-producing streptococci; bacteriocin-like inhibitory substances such as *S. salivarius* [7]; and streptococci that combine several of these mechanisms of action such as *S. oralis subsp. dentisani* (abbreviated to *S. dentisani*), which produce bacteriocin and have arginolytic activity [8,9].

Down syndrome (DS) is the most common chromosomal anomaly, with an estimated prevalence of one case for every 800 live births [10]. This chromosomal disorder is characterized by a varying degree of intellectual disability and numerous systemic disorders including cardiac, hematological, and endocrine abnormalities [10]. The most relevant oral manifestations of DS are macroglossia, malocclusions, delayed tooth eruption and periodontal disease [11]. It has been suggested that the incidence of dental caries in children and adolescents with DS is lower than in the non-syndromic population [12,13,14,15], although the quality of the available evidence does not allow for a definitive inference [16]. Based on this argument, the Special Care Dentistry Unit of the University of Santiago de Compostela (Spain) analyzed the oral flora of a patient group with DS with no evidence of caries to search for bacterial strains with antagonistic effects against *S. mutans*, which helped identify a new bacterial species called *Streptococcus downii* sp. nov. (abbreviated to *S. downii*) [17].

*S. downii* is a bacteria of the *Streptococcus* genus whose genetic and taxonomic analysis places it within the oral group, the *Streptococcaceae* family and the *Lactobacillales* order. *S. downii* is an alpha-hemolytic, Gram-positive coccus that differs biochemically from the closely related *Streptococcus* species due to the production of α-galactosidase, β-galactosidase and N-acetyl-β-D-glucosaminidase, and by the absence of arginine dihydrolasedesiminase and IgA1 protease. The most attractive characteristic of *S. downii* due to its potential for preserving oral health is that it inhibits the growth of *S. mutans* and certain periodontal pathogens such as *Aggregatibacter actinomycetemcomitans*, both in plaque growth [17] and in an *in vitro* biofilm model [18]. Although its mechanism of action has not been definitively clarified, the genomic analysis of *S. downii* has revealed the presence of putative genes of the family of bacteriocin IIb lactobinA/cerein 7B (data not shown).

The aim of this study was to determine the distribution of *S. downii* in the oral cavity of individuals with DS and in the general population and to investigate whether there is a clinical-microbiological correlation in terms of the prevalence of caries and periodontal disease.

## 2. Materials and Methods

### 2.1. Design of a Specific PCR for the Bacteriocin Operon

**Figure 1:**
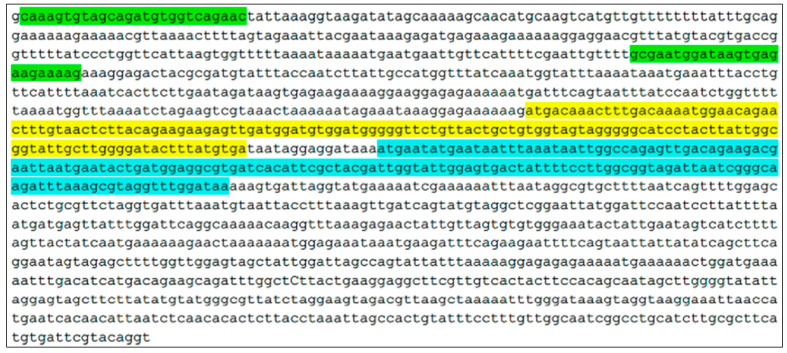
DNA sequence of *S. downii* indicating the position of the primers (marked in green) and the two open reading frames of the encoded bacteriocins in this fragment (marked in blue and yellow).

In the *S. downii* MZNX00000000.1 sequence, we selected the contig that contained the sequence corresponding to the bacteriocin. We designed specific primers for the bacteriocin operon (class IIb lactobin A/cerein 7B family) with the Primer-BLAST program (https://www.ncbi.nlm.nih.gov/tools/primer-blast/ (accessed on 10 June 2017). The sequences of the starters were as follows: *S. downii*-FORWARD CAAAGTGTAGCAGATGTGGTCAGAAC and *S. downii*-REVERSE CTTTTCTTCTCACTTATCCATTCGC (Figure 1).

**Figure 2:**
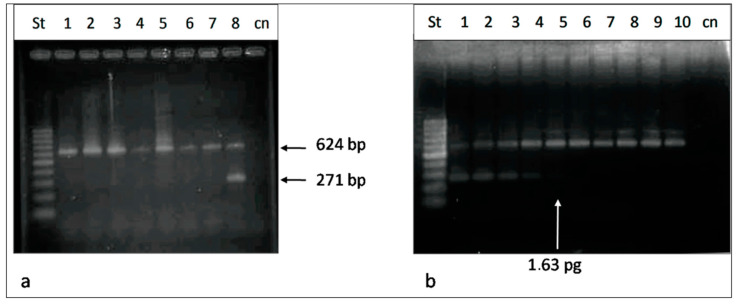
(**a**) Specificity assay using primers specific for the bacteriocin operon of *Streptococcus downii* (St.1: *S. mitis*; St.2: *S. salivarius*; St.3: *S. dentisani* 8313; St.4: *S. dentisani* 8312; St.5: *S. mutans*; St.6: *S. sobrinus*; St.7: *S. sanguinis* DSTIZ 20567; St.8: *S. downii*). (**b**) Sensitivity assay using primers specific for the bacteriocin operon of *Streptococcus downii* (St.1: 460,000 copies, 1 ng of bacterial DNA; St.2: 92,000 copies, 2 × 10^−1^ ng; St.3: 18,400 copies, 4 × 10^−2^ ng; St.4: 3680 copies, 8 × 10^−3^ ng; St.5: 736 copies, 1.6 × 10^−3^ ng; St.6: 147.2 copies, 3.2 × 10^−4^ ng; St.7: 29.44 copies, 6.24 × 10^−5^ ng; St.8: 5.888 copies, 1.28 × 10^−5^; St.9: 1.1776 copies, 2.56 × 10^−6^; St.10: the DNA of *S.downii* was not included).

Through partial magnification of the gene rRNA 16S, we performed a specificity assay on DNA from seven bacterial species related to *S. downii* (*S. mitis*, *S. salivarius*, *S. dentisani* 8313, *S. dentisani* 8312, *S. mutans*, *S. sobrinus* and *S. sanguinis* DSTIZ 20567), including an internal reaction control. We showed that the designed primers were specific for *S. downii* and amplified a region included in the bacteriocin operon of 271 bp (Figure 2a).

With a fixed quantity of bacterial DNA (1 ng), we performed serial dilutions of the *S. downii* DNA (dilution factor range: 5–390,625). The lower limit of detection was approximately 1.63 pg, which corresponded to 736 genomic equivalents (GEs) of *S. downii* DNA (Figure 2b).

### 2.2. Validation of the Specific PCR

To validate the quantitative PCR using specific Taqman probes for detecting and quantifying a region of the gene of bacterial RNA 16S and of a sequence adjacent to a bacteriocin gene of *S. downii*, we analyzed four samples of supragingival dental plaque seeded with *S. downii* at 3000 colony-forming units (CFU)/mL. To process the samples, we first centrifuged them at 13,000 rpm for 15 min. We used 250 μL for the resuspension of the bacteria and the DNA extraction using the QIAamp DNA Blood Mini Kit (Qiagen, Germany). The column’s elution volume was 100 μL; the DNA concentration was therefore 30 GEs of *S. downii* per μL. The amplification was performed in real time using a duplicate for each sample. To perform the assay, we used 2 μL per sample. To proceed with the quantification, we included a curve of the DNA pattern for *S. downii* obtained with the same purification kit. The curve included six determinations corresponding to serial dilutions at 1/5 starting at 92,000 GEs and ending at 29 GEs.

Table S1 shows the mean quantification cycle (Cq) values and the mean concentrations obtained for the target (bacteriocin) and control genes (16S rRNA) estimated based on the pattern curve obtained by diluting the bacterial DNA of *S. downii* quantified by fluorometry. The detected GEs of *S. downii* were similar to the CFUs seeded in the samples, with a mean concentration of 24 GE/μL.

### 2.3. Study Population

We designed an observational, cross-sectional, case-control study that was approved by the Research Ethics Committee of Santiago-Lugo, Spain (registration code: 2018/510). We established a convenience group of cases consisting of 200 white male individuals with DS recruited consecutively from the attendees of educational and occupational therapy centers of Galicia (Spain) from 2018 to 2019. For the recruitment and to facilitate the collaboration of the individuals with DS in a known setting, the researchers travelled to the centers of A Coruña, Lugo, Pontevedra, Ferrol, Ourense, Vigo and Santiago de Compostela.

The applied inclusion criteria were as follows: confirmed genetic diagnosis of DS, age >3 years (coinciding with the age of complete eruption of the temporary dentition), presence in the mouth of Ramfjord teeth [19] or of their adjacent teeth (of the corresponding temporary teeth, if applicable), sufficient collaboration to allow for intraoral examination and sampling and voluntary participation in the study (informed consent signed by the participants or their legal representatives). We excluded individuals with chronic disease, medication consumption and previous procedures (e.g., radiation therapy of the head and neck) that reduced salivary flow, and we excluded those who were administered antibiotics in the previous 3 months or who regularly used antiseptic mouthwash in the previous week.

Applying the same criteria, we established a control group (CG) consisting of 100 non-syndromic individuals among the companions of the patients with DS.

### 2.4. Oral Health Condition and Collection of Samples

Each participant’s age was recorded, and their oral health state was assessed. Due to the potential limitations in some of the patients’ collaboration, the duration of the examination was minimized by limiting it to the six Ramfjord teeth [19]. The following clinical variables were recorded: index of visible dental plaque around the tooth [20]; presence of active caries applying the International Caries Detection and Assessment System [21]; gingivitis, when the probing depth was <4 mm and bleeding on probing detected in more than two locations [22]; and periodontitis, when the probing depth was ≥4 mm in at least one location [23].

In total, two samples of supragingival dental plaque were collected from each patient using a conventional metal curette (Hu-friedy, Frankfurt, Germany). The curette was inserted into an Eppendorf tube containing 50 μL of saline solution and transported to the laboratory, where it was stored at −20 °C until processing. The samples were unfrozen at room temperature and transferred to a 2-mL tube. The process of centrifugation and DNA extraction was then started, as described in Section 2.2. In all of the sample processing series for the DS group and CG, we included two samples of positive controls contaminated with *S. downii* at 3000 CFU/mL.

### 2.5. Detection of S. downii in Metagenomes of Supragingival Plaque of the “Human Microbiome Project”

To evaluate the prevalence of *S. downii* in the supragingival plaque of healthy individuals, we compared the whole genome and the regions related to *S. downii* bacteriocins against metagenomes of supragingival plaque of the Human Microbiome Project [24]. Of the 217 metagenomes initially identified, we selected 127 that were assembled, generated by whole genome sequencing (WSG) techniques. We calculated the average nucleotide identity based on MUMmer (ANIm) between the *S. downii* genome and each of the selected metagenomes, applying the NUCmer tool [25] using pyANI (v0.2.7) [26], as previously described [27]. To confirm the presence of orthologous genes related to bacteriocins, we used the Blast reciprocal best hits (BRBH) method [28].

## 3. Results and Discussion

### 3.1. Prevalence of S. downii in the Oral Cavity of Individuals with Down Syndrome and of the General Population

Bacterial DNA was detected in all of the analyzed samples from the DS group and CG. The range of the bacterial 16S rRNA was 12.15–24.90 Cqand 4.29 × 10^3^–8.90 × 10^6^ GE/μL. We detected the specific gene of the *S. downii* bacteriocin in an individual with DS (prevalence of 0.5%), with values of 34.52 Cq and 13.0 GE/μL. We also detected the specific gene of the *S. downii* bacteriocin in an individual in the CG (prevalence of 1%), with values of 34.78 Cq and 4.93 GE/μL. In all of the positive contaminated control samples, we identified the specific gene of the *S. downii* bacteriocin.

Bacteriocins are small peptides or proteins of bacterial origin that inhibit the growth of other bacteria, favoring the colonization of the producing species [29]. Class IIb bacteriocins consist of two peptides (<10 kDa), which act by increasing the porosity of the target cells’ membrane [30]. *S. downii* produces a class IIb bacteriocin lactobinA/cerein 7B that is thermostable and resistant to the action of proteinase K [31].

Using primers specific to this bacteriocin’s operon, the detected prevalence of *S. downii* in this study was ≤1%, both in the DS group and in the general population. This bacteriocin was identified in the two strains of *S. downii* deposited in the Spanish Type Culture Collection (*Colección Española de Cultivos Tipo*, CECT) and in the Swedish collection (Culture Collection University of Gothenburg; access numbers CECT:9732T and CCUG:73139T). This could represent a limitation of the study, given that the prevalence of *S. downii* could have been underestimated, if it is confirmed that not all strains of *S. downii* express the same bacteriocins or the same bacteriocin-related gene clusters, as occurs with *S. dentisani* [32].

The sequencing of the 16S rRNA gene has been the most widely used genetic marker by far in recent years for studying bacterial phylogeny and taxonomy, although it is not exempt from a number of limitations such as sequence similarity between different species of the *Streptococcus* genus [33]. Careful interpretation of the results of the distribution of other streptococci with *S. mutans* inhibitory activity such as *S. dentisani* is required. *S. dentisani* could be overestimated, given that it has been identified in most metagenomes of dental plaque stored in the Human Microbiome Project [9] and in all of the samples of saliva and dental plaque analyzed from various geographical locations worldwide [34]. To increase the reliability of the taxonomic assignation at the *Streptococcus* species level in the saliva microbiota, it has been suggested that matrix-assisted laser/desorption ionization time-of-flight mass spectrometry analysis is an accurate and consistent method [35]. The use of tools such as the web server BAGEL4 enables the (meta-) genomic analysis of bacterial DNA sequences to identify bacteriocins [36] and has already been incorporated among the methods for analyzing bacteriocin clusters of *S. dentisani* [32].

### 3.2. Prevalence of S. downii in Metagenomes of Supragingival Plaque of the Human Microbiome Project

Of the 127 selected metagenomes, only one obtained an ANIm with ≈95% homology (metagenome SRS013533), which had the highest nucleotide identity with *S. downii*. In the *S. downii* genome, we detected the presence of two open reading frames (ORFs) that coded class IIb lactobiocin A/cerein 7B bacteriocins and nine ORFs that coded accessory proteins to these bacteriocins (Table 1). When analyzing the presence of these genes in the 127 selected metagenomes, we detected 100% homology in metagenome SRS013533, along with high homology of the ORFs related to accessory proteins to this bacteriocin (Table S2). Although we obtained a high degree of homology of the genes related to bacteriocins in another four metagenomes, the ANIm value was <94% in all of them. This result allows us to conclude that the prevalence of *S. downii* in the metagenomes of supragingival plaque of the Human Microbiome Project was 0.7%, which agrees with the results using the PCR designed for the operon of bacteriocin (class IIb lactobin A/cerein 7B family).

Other potential methodological limitations are those derived from the metagenome-genome comparison and the small size of the detected sequences of the genes related to bacteriocins. The recommended cut-off point of 70% DNA-DNA hybridization (DDH) for species delineation corresponds to 95% ANI [27,37,38], which was what we detected in metagenome SRS013533. Although the ANI represents the percentage of identity of preserved genes between two strains, the threshold suggested in recent studies for the genome-genome comparison has been equally useful for the metagenome-genome comparison [39]. Although the size of the aligned sequence in the search for genes related to bacteriocins was small, it was close to the criterion proposed by other authors [40].

### 3.3. Clinical Results of Individuals with Down Syndrome and of the General Population

The results of the clinical intraoral examination are detailed in Table 2. Although this was not a primary objective of the study *per se*, it was essential for establishing a clinical-microbiological correlation in terms of the prevalence of caries and periodontal disease. This correlation obviously could not be established due to the low detected prevalence of *S. downii*.

Visible dental plaque was detected in 60.0% and 76.0% of the DS group and CG, respectively. For those younger than 30 years, the rates were 61.5% and 72.1%, respectively. Among those older than 30 years, 51.6% and 90.4% of the DS group and CG had dental plaque, respectively. The prevalence of caries was 40.5% and 51.0% among the DS group and CG, respectively. For those younger than 30 years, the rates were 39.0% and 53.1%, respectively. Among those older than 30 years, 48.3% and 42.8% of the DS group and CG had caries, respectively. Some 71.5% of the DS group and 66.0% of the CG had gingivitis. For those younger than 30 years, the rates were 68.0% and 67.1%, respectively. Among those older than 30 years, 90.3% of the DS group and 61.9% of the CG had gingivitis. The diagnostic criteria for periodontitis were met by 35.5% and 32.0% of the DS group and CG, respectively. For those younger than 15 years, periodontitis was detected in 14.5% of the DS group and in none of the CG. Among those younger than 30 years, the rates were 30.7% and 31.6%, respectively. Among those older than 30 years, 61.2% and 33.3% of the DS group and CG had periodontitis, respectively.

Although it has been speculated that the combination of poor control over dental plaque and a deficient immune system might promote the development of caries in individuals with DS, the present series recorded a lower prevalence of caries in the DS group than in the CG, confirming the results of previous studies [12,13,14,15]. It has been argued that this finding could be due to delayed tooth eruption, microdontia, tooth agenesis and the presence of diastemas [41]. The bacterial flora plays a fundamental role in the etiopathogenesis of caries, and it has been shown that children and adolescents with DS have a lower density of *S. mutans* in saliva samples than nonsyndromic controls [42,43]. Additionally, the molecular typing of *S. mutans* using arbitrarily primed PCR revealed that the strains that colonize the tooth surface have different, presumably less cariogenic profiles in DS than in non-syndromic controls [44].

In the present series, gingivitis and periodontitis were more prevalent in the DS group than in the CG, confirming that gingivitis and periodontal disease occur more frequently in individuals with DS, have an earlier onset and are more severe than in the general population [45,46,47]. It has been suggested that the development of periodontitis in these patients is due to a specific subgingival microbiota [48], along with the host’s abnormal, syndrome-inherent immune response [49].

Recent studies that applied next-generation sequencing have shown substantial differences in the composition of the oral microbiome between patients with DS and non-syndromic individuals, with a significantly lower diversity of species (i.e., alpha diversity) and an increased presence of periodontal pathogens in DS [50]. In individuals with DS, the microbiome differs significantly between those who have an oral condition (e.g., periodontal disease) and those who do not [51]. The influence of bacteria with anticaries and antiperiodontitis potential such as *S. downii* on the composition and homeostasis of the oral microbiome in individuals with DS is still unknown.

It has been suggested that, for individuals with DS and periodontal disease, non-surgical periodontal therapy is effective in terms of clinical parameters; unlike with euploid individuals, however, this therapy does not cause a significant reduction in red-complex bacteria levels in deep periodontal pockets [52]. Accordingly, these authors suggested that, in individuals with DS, the results of mechanical periodontal treatment might be improved if adjuvants are used to reduce periodontal pathogen counts [52]. The fight against antibiotic resistance has become a priority health action, in which infection prevention and a more restrictive antibiotic policy are essential [53]. Accordingly, there has been growing interest in a number of alternative strategies to antibiotics in the dental setting, strategies that do not share the adverse effects of antibiotics, such as antimicrobial photodynamic therapy [54] and the use of probiotics to re-establish oral homeostasis [55]. The interest in detecting *S. downii* lies in its antibacterial activity against a number of cariogenic species and certain periodontal pathogens [17,18], which makes *S. downii* a potential probiotic.

## 4. Conclusions

Applying a specific PCR for the bacteriocin operon (class IIb lactobin A/cerein 7B family) of *S. downii*, the detection rate of this novel streptococcal species in the supragingival dental plaque was ≤1%, both in Down syndrome and in the general population. A clinical-microbiological correlation could therefore not be established.

## Figures and Tables

**Table 1:** Main genes related to bacteriocins present in the genome of *S. downii*.

ORF id	Contig	ORF Start	ORF End	Strand	Description
gene_00016	contig_1	13439	13582	+	Class IIb bacteriocin, lactobin A/cerein 7B family
gene_00017	contig_1	13598	13756	+	Bacteriocin-type signal sequence domain-containing protein
gene_00019	contig_1	14153	14308	+	Class IIb bacteriocin, lactobin A/cerein 7B family protein
gene_00020	contig_1	14347	14559	+	Bacteriocin immunity protein
gene_00022	contig_1	14853	15062	+	Bacteriocin immunity protein
gene_00024	contig_1	15745	17898	+	Peptide cleavage/export ABC transporter
gene_00402	contig_6	58328	58624	−	Bacteriocin immunity protein
gene_00657	contig_6	307826	309934	−	Bacteriocin-associated integral membrane family protein
gene_00673	contig_6	326623	326919	−	Bacteriocin immunity protein
gene_01111	contig_8	277351	277698	−	Thiol reductase thioredoxin
gene_01818	contig_9	605338	606096	+	Bacteriocin ABC transporter ATP-binding protein

**Table 2:** (**a**) Clinical Characteristics of the Study Group with Down syndrome. (**b**) Clinical Characteristics of the non-syndromic Control Group.

**(a)**					
**Age** **(Years)**	**Dental Plaque** **(*n*)**	**Caries** **(*n*)**	**Gingivitis** **(*n*)**	**Periodontal** **Disease (*n*)**	**Total** **(*n*)**
0–5	1	2	0	0	13
6–10	12	7	9	2	20
11–15	13	2	15	6	22
16–20	28	19	32	12	42
21–25	25	14	26	13	34
26–30	25	22	33	19	38
31–35	7	6	13	9	14
36–40	7	6	8	3	10
41–45	2	2	4	4	4
46–50	0	0	1	1	1
51–55	0	1	2	2	2
56–60	0	0	0	0	0
Total	120	81	143	71	200
**(b)**					
**Age** **(Years)**	**Dental Plaque** **(*n*)**	**Caries** **(*n*)**	**Gingivitis** **(*n*)**	**Periodontal** **Disease (*n*)**	**Total** **(*n*)**
0–5	1	1	1	0	3
6–10	9	7	8	0	11
11–15	14	9	14	0	16
16–20	10	9	9	9	10
21–25	15	15	15	15	28
26–30	8	1	6	1	11
31–35	2	1	2	1	2
36–40	12	6	7	2	14
41–45	3	1	3	3	3
46–50	1	1	1	1	1
51–55	0	0	0	0	0
56–60	1	0	0	0	1
Total	76	51	66	32	100

## Data Availability

Data available upon request due to restrictions. The data presented in this study are available on request from the corresponding author. The data are not publicly available due to privacy issues.

## Supplementary Materials

The following supporting information can be downloaded at: https://www.mdpi.com/article/10.3390/microorganisms10061098/s1, Table S1. Quantification cycle (Cq) value and genome equivalent of S. downii (GE)/μL for the target (bacteriocin) and control genes (16S rRNA) of supragingival dental plaque samples seeded with S. downii at 3000 CFU/mL. Table S2. Results of the analysis using Blast reciprocal best hits (BRBH) of the genes related to bacteriocins present in *S. downii* in the metagenome SRS013533.
