# Supplementary material for: Molecular Detection of Streptococcus downii sp. nov. from Dental Plaque Samples from Patients with Down Syndrome and Non-Syndromic Individuals
Source: Microorganisms. 2022 May 26;10(6):1098. doi: 10.3390/microorganisms10061098 (PMC9227364; doi:10.3390/microorganisms10061098)
Supplement: Supplementary file 1 [file microorganisms-10-01098-s001.zip › Supplementary.pdf]

Table S1. Quantification cycle (Cq) value and genome equivalent of *S. downii* (GE)/uL for the target (bacteriocin) and control genes (16S rRNA) of supragingival dental plaque samples seeded with *S. downii* at 3000 CFU/mL.

	**Gene 16S bacterial rRNA**		**Bacteriocin gene of *S. downii***	
**Sample**	Cq Mean	GE/µL	Cq Mean	GE/µL
1. Dental plaque	14.05	555,000	32.40	17
2. Dental plaque	21.12	13,915	21.06	41
3. Dental plaque	16.69	140,250	31.89	24
4. Dental plaque	18.31	60,350	32.79	14

Table S2. Results of the analysis using Blast reciprocal best hits (BRBH) of the genes related to bacteriocins present in *S. downii* in the metagenome SRS013533

Query	Hit	% Identity	Alignment length	Evalue	BitScore	Metagenome
contig_1: 13598-13756	SRS013533_PGA_scaffold_29144	96.835	158	3.21E-70	265	SRS013533
contig_1: 14153-14308	C5197739	100	29	0.00000074	54.7	SRS013533
contig_1: 15745-17898	SRS013533_PGA_scaffold_37401	96.183	131	5.18E-54	215	SRS013533
contig_8: 277351-277698	SRS013533_PGA_scaffold_39169	100	48	4.96E-17	89.8	SRS013533
